# Computer-assisted coloring and illuminating based on a region-tree structure

**DOI:** 10.1186/2193-1801-1-1

**Published:** 2012-03-06

**Authors:** Renata Nascimento, Fabiane Queiroz, Allan Rocha, Tsang Ing Ren, Vinicius Mello, Adelailson Peixoto

**Affiliations:** 1 Department of Mathematics, PUC-Rio, Rio de Janeiro, Brazil; 2 Department of Computer Science, UFPE, Pernambuco, Brazil; 3 Department of Computer Science, PUC-Rio, Rio de Janeiro, Brazil; 4 Institute of Mathematics, UFAL, Maceio´, Brazil; 5 Institute of Mathematics, UFBA, Salvador, Brazil

**Keywords:** 2D drawing, Region-tree, Illumination, Colorization, Animation

## Abstract

Colorization and illumination are key processes for creating animated cartoons. Computer assisted methods have been incorporated in animation/illustration systems to reduce the artists' workload. This paper presents a new method for illumination and colorization of 2D drawings based on a region- tree representation. Starting from a hand-drawn cartoon, the proposed method extracts geometric and topological information and builds a tree structure, ensuring independence among parts of the drawing, such as curves and regions. Based on this structure and its attributes, a colorization method that propagates through consecutive frames of animation is proposed, combined with an interpolation method that accurately computes a normal mapping for the illumination process. Different operators for curve and region attributes can be applied independently, obtaining different rendering effects.

## Introduction

Since the 1960s, computers became a great ally in the production of animated films, contributing to several areas that goes from the creation to the final touches [1].

In the conventional production of an animation sequence, first of all is necessary a representation of frame by frame [2]. Even today, this technique is used in the production of 2D animated cartoons in which each frame is represented by a free-hand sketch.

Although assisted animation accelerate the procedure, it still has many limitations which drives one of the largest areas of research in computer graphics: Computer Animation.

In the conventional process of computer assisted cartoon animation, the entire process for the production is very costly and may even take months for a short animation to be finished. Among the biggest challenges of an automated solution, the production of inbetweening and frame colorization as mentioned by [3] is of great importance.

Two important, although tedious, steps in the production of animated cartoons are colorization and illumination. The colorization process transfers the colors from a single frame to the subsequent frames. The most common colorization approaches [4,5] use structures containing topological information of the drawing, like regions, curves, and graphs, called topological structures.

Although these topological structures perform well in the colorization process, they are not commonly exploited in the illumination process of the animation. The illumination process calculates the interaction of the cartoon with light present in a 3D environment, and is an important task in cartoon rendering, since it helps to produce different effects and styles in the animation sequence.

### Contributions

This paper presents three main contributions. The first one is a new region-tree structure that explores local spacial information in a single frame. The second contribution is a method based on the region-tree to illuminate the objects, by approximating lighting on 2D drawings using a direct and sphere-preserving interpolation. Third, we propose a recursive tracking method for color transfer based on the region-tree. This new approach improves more effective associations of regions of two consecutive frames that can be retrieved through a recursive analysis of previous frames.

### Related work

#### Regions representation

An efficient image region representation has been sought in different ways. A common approach is the use of an hierarchical structure which clusters regions at different scales to obtain the representation of images in various resolutions [6-8]. These techniques are ideal in processes such as image compression. However, in images like cartoons this structure could be simplified, since simpler inherent relationship of regions, such as inclusion and adjacency relations, will be necessary during the cartoon illumination and colorization.

#### Illumination

In some works [9,10], automatic normal estimation methods is computed directly from the image. But these techniques calculate the normals from the shading information of the images. They are not suitable for cartoon, because in our input cartoon we have neither normal nor shading. Some techniques to illuminate 2D data provide iterative tools to reconstruct 3D models from available bi-dimensional data [11,12], followed by traditional 3D illumination [13]. Johnston [14], observes that, even though these methods provide useful results, they may not be suitable to cartoon rendering. Therefore, the author [14] proposes a semi-automatic image-based technique to approximate surface normals directly in 2D drawings while avoiding 3D reconstruction. How-ever, this technique does not enable different kinds of interpolation since it does not have a relationship between curves and regions. Besides it obtains an approximated normal propagation to calculate the normal map. Recently, depth-propagation proposed by Sy´cora et al. [15] obtain correct initial normal map of cartoons, but the interpolation itself is computed later, by using the method proposed by Johnston [14]. In our work we explicitly calculate the normal vector accurately and guarantees smoothness for a coherent illumination. The region- tree flexibility allows different visual effects since it is possible to choose which curve contributes to the regions interpolation process.

#### Colorization

Segmentation techniques are generally used to extract topological structures, and employ tracking operations to check the time coherence between consecutive frames, enabling a computer-assisted colorization [4,16-18]. Although these techniques provide the colorization to animating cartoons, these approaches do not exploit essential information such as curves and interior regions for the illumination process. Garcia et al. [19] propose a strategy for region matching which deals with the curve closeness issue. Since the matching is rather geometric, the method robustness could be affected Sy´cora et al. [15] proposes a depth-propagation to handle cartoon animations resulting in automatic colorization. However, this approach does not handle occlusion during tracking. In this work, we propose a topological approach as a way to increase the robustness of region tracking enabling it to handle occlusions.

The remainder of the paper is structured as follows. Section 2 formulates our proposed structure, the region-tree, that represents cartoon geometrical and topological elements. Section 3 describes the proposed illumination method. Section 4 presents a recursive colorization method based on region-tree. Section 5 presents some experimental results and Section 6 concludes.

## Region-tree

This section describes a topological structure, which contains the topological and geometric information of cartoons objects. Similar to several other methods, we first apply a segmentation stage to extract a set of curves and regions [4]. In a later stage, we explore the relationship between regions and curves to construct the data structure *region-tree*.

### Curves and regions

Essentially, a cartoon could be represented by their curves and regions. The traces or outlines of the cartoon define the curves, while the interior of closed curves define the regions. We use the operator sets described by Bezerra et al. [4] to construct the set of regions and curves that will compose our region-tree structure. Those operations includes: *skeletonization *and *region detection*.

**Figure 1:**
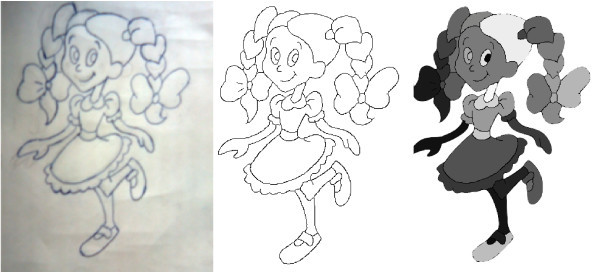
**Input hand drawing cartoon (left)**. Skeletonization(middle). Region detection (right).

*Skeletonization *or *thinning *is the extraction process of a linear subset of images' lines, which emphasizes geometrical and topological properties of the shape. The main idea is simplify the objects representation without changing its topology. Therefore, the skeletonization algorithm removes redundant points by eroding the image while preserving the lines connection and hence the topology of the drawing (see Figure 1 middle). The final points constitute the skeleton. In this work, the Zhang-Suen algorithm [20] was used.

*Region detection *consists on performing the segmentation of the thinning image identifying each of its curves and regions. Each curve is defined by a sequence of pixels from the skeleton. In our structure, each region is defined by three informations: its boundary (external curve), its interior points (the region itself), and its internal curves. This structure will help us to calculate more efficiently the relations of inclusion and adjacency between regions in our region-tree. To extract each region, we first compute the internal area by assigning a label to each connected internal pixel. So, each connected component of the image will have the same label and will define the interior of a region. A flood-filling algorithm [21] is performed to make such labeling (see Figure 1 right).

**Figure 2:**
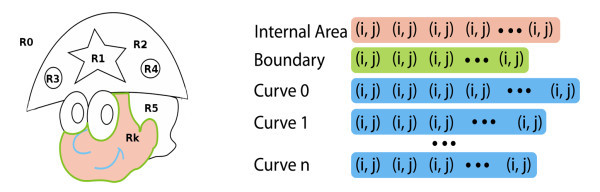
**Region Structure: Each region *R_k _*is composed by: a set of internal pixels, labeled by *Internal Area*; a set of boundary points, labeled by *Boundary *and a set of internal curves, labeled by *Curve 0 *to *Curve n***.

To extract the curves of a region (boundary and internal curves) we will sort the skeleton pixels, introducing a polygonal representation to the curves. Although the internal curves are not necessary for a region definition, they have a great value in the representation since they provide a variety of details giving expressivity to the drawing. A polygonal representation for each regions curve is obtained by a chain-code algorithm [22] with 8-connected neighborhood. After this stage, for each region is possible to access immediately its internal area (set of labeled pixels), its boundary curve and the set of internal curves both in a polygonal representation (see Figure 2).

### Region-tree generation

Now we describe our proposed region-tree structure. Usual topological structures are used in colorization methods to track regions of consecutive frames in the animation process [4,16-18]. Those structures explore the relationships of adjacency between regions to preserve the temporal coherence. However, they do not explore some local information details in a single frame, such as inclusion relation between regions inside the drawing. In order not only to provide time relational coherence between frames, the structure proposed in this paper aims to also allow exploring each images component individually. The possibility of navigation between the regions structures and their included regions allows treating the components one by one enabling a wide variety of effects in the drawings.

**Figure 3:**
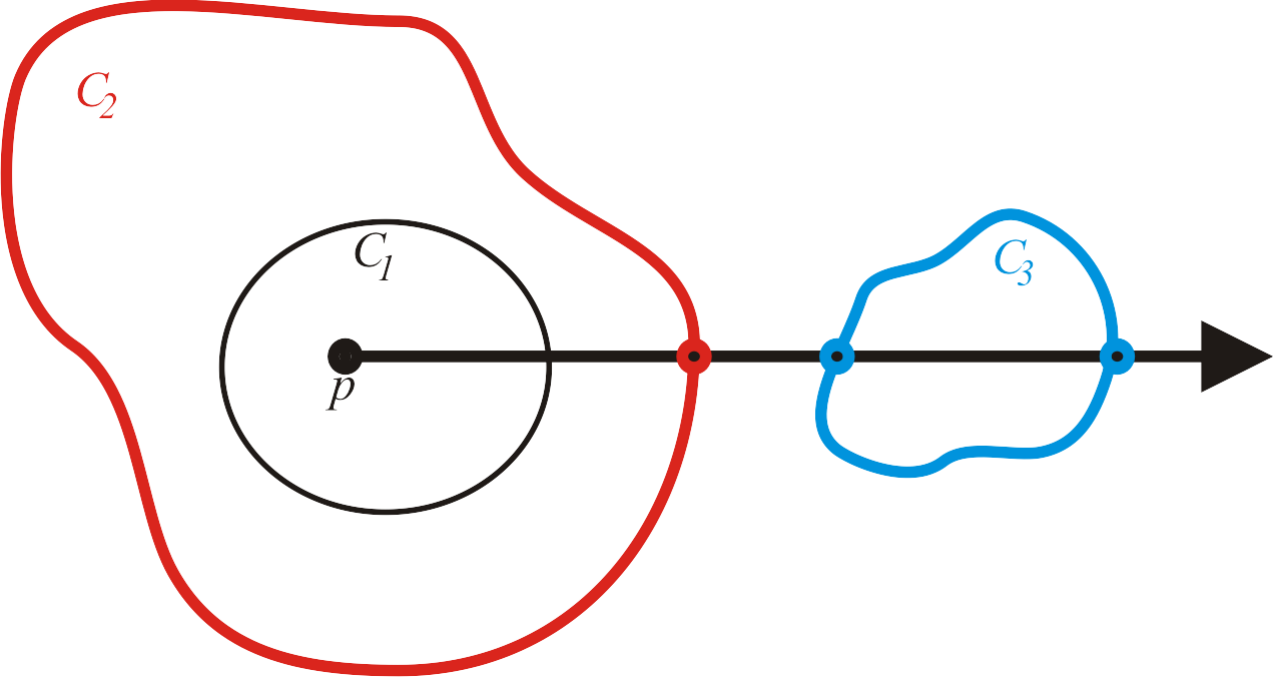
**Since the ray casted from *p *intersect *C*_2 _one time, *C*_1 _is contained in *C*_2_**. On the other hand, the ray intersect *C*_3 _an even number of times, so *C*_1 _is not contained in*C*_3_.

The structure proposed is defined as a tree, where each node contains a region *R_i_*, and the set of its descendant nodes represents the internal regions (subsets). Our strategy to decide whether a region bounded by a curve *C*_1 _is contained in another region bounded by a curve *C_i _*is based on the theorem of Jordan [23]. Thus, given a point *p *inside the curve *C*_1_, we cast a ray from *p *and find the number *n *of intersections of the ray with the curve *C_i_*. If *n *is odd, then *C*_1 _is contained in *C_i _*(see Figure 3).

**Figure 4:**
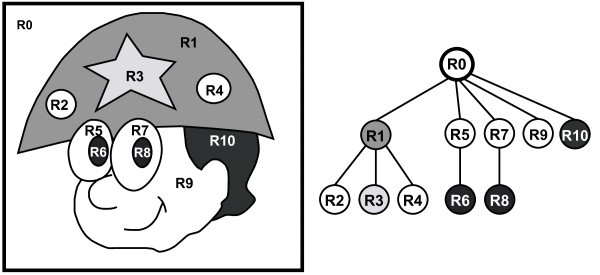
**Region-Tree Structure: Each node represents one region in the image**. In this example, the node *R*3 is a child node of *R*1. So the region *R*3 is an internal region of *R*1.

Besides being a simple implementation structure, it contains more information than usual topological structures (see Figure 4), such as the ones described by Sy´cora et al. [15].

In Section 3, we present how the region-tree structure assists in the illumination process of a 2D drawing. Its advantage stems from the possibility of dealing individually, and in a consistent way, each images region allowing also process them in parallel.

The region-tree structure provides total control in the access of each regions component as boundary, internal curves, internal pixels, adjacent regions and contained regions. This enables separate attributes for each cartoon region: material properties, color, transparency, texture, and others. Analogously, we can define an attribute to each curve, or group of curves: color, line style, width, and others. Regions attributes can be stored as frame buffers allowing the data to be processed directly on the GPU.

In the context of this work, the normal vectors to the curves and regions perform a role of great significance. The inference of a normal field to the image, make it possible for any illuminations model providing three-dimensional effects on the cartoon. The next section describes this attribute in detail.

## Illumination and normal mapping

In this work, we propose a novel formulation for the normal field inside each region. This normal field enables the application of illumination effects, like Phong model, providing 3D impression to the cartoon. Our normal definition is accurate and sphere-preserving, which leads to smooth and coherent illumination.

### Normal field of the curves

For each region, we first compute the normal field of the boundary [4]: given two consecutive boundary points *p*_1 _= (*x*_1_, *y*_1_) and *p*_2 _= (*x*_2_, *y*_2_), we compute the tangent vector *v *= (*x*_2 _*− x*_1_, *y*_2 _*− y*_1_). So, the normal vector at *p *is given by *n *= (*y*_2 _*− y*_1_, *x*_1 _*− x*_2_, 0).

The coordinate *z *= 0 indicates that the image plane is the projection plane relative to the viewer and the curve belongs to the object's silhouette.

Since we are working in an 8-connected neighborhood, the normal vector obtained above is restricted to these eight directions. This constraint has as consequence a low quality at the illumination effect. Thus, if a point *p_i _*has normal *v_i_*, a smoothing process is obtained by applying a discrete Gaus-sian filter to the normal *vi′*. The new normal vector *vi′ *is

$$\nu_{i}^{\prime }=\frac{\left(\nu_{i-2}+4\nu_{i-1}+6\nu +4\nu_{i+1}+\nu_{i+2}\right)}{16},$$

where *v_i−_*_2_, *v_i−_*_1_,*v_i_*_+1 _and *v_i_*_+2 _are the normal vectors of the *p_i _*neighbors. The normal vectors of the internal curves of the region are computed in a similar way.

### Normal field of the interior

The normal field of the interior of the region is interpolated from the boundary normal field. Our proposed formulation is described in the continuous domain:Let *C *be a closed curve parameterized by arc-length, *C *(*s*) = (*x*(*s*), *y*(*s*)), and let *R *be a region delimited by *C*. The normal vector *n*(*s*) at each point of *C *(*s*) is given by:

$$n\left(s\right)=\left(nx\left(s\right),ny\left(s\right),0\right)=\left(y^{\prime }\left(s\right),-x^{\prime }\left(s\right),0\right).$$

Our task is to compute a 3D normal

n(p)=(nx(p),ny(p),nz(p)),

for each point *p *= (*x, y, z*) in ℝ.

First of all, we compute the components *n_x _*and *n_y_*. This is done by integrating the normal contributions along the curve *C*, where the contribution of each point *C *(*s*) is weighted by the inverse square distance from *p *to *C *(*s*). So, for each *p ∈ R*, *n_x _*(*p*) and *n_y _*(*p*) are obtained by:

$$\begin{array}{ll} n_{x}\left(p\right) & =\frac{\int_{C}\frac{n_{x}\left(s\right)ds}{{\left|p-C\left(s\right)\right|}^{2}}}{w\left(p\right)},\;\\ n_{y}\left(p\right) & =\frac{\int_{C}\frac{n_{y}\left(s\right)ds}{{\left|p-C\left(s\right)\right|}^{2}}}{w\left(p\right)},\;\\ & \; \end{array}$$

where

$$w\left(p\right)=\int_{C}\frac{ds}{{\left|p-C\left(s\right)\right|}^{2}}$$

The next step is to calculate the *n_z _*component. This step is similar to [14]. We consider that the normal field we want to reconstruct is over a surface that rises smoothly from the curve *C (s) *contained in the image plane (where *n_z _= *0). This normal field points toward the viewer, so the *n_z _*component is positive. In addition, *nz *should be calculated so that the normal vector field is normalized. So, the *nz *component is obtained by:

$$n_{z}\left(p\right)=\sqrt{1-n_{x}{\left(p\right)}^{2}-n_{y}{\left(p\right)}^{2}},$$

If the region has internal curves, the normal vectors of theses curves may be used to compose the final normal field of the interior of the region during the interpolation process presented above. If these internal curves are not considered during the interpolation process, only the boundary curves will affect the normal field of the region.

### Normal field analysis

We now do an analysis of the surface "generated" by our normal interpolation method. Is it smooth? How it behaves over its domain (the image plane)? First of all, we demonstrate that if *C *is a circle our interpolation technique provides exactly the normal field of a sphere.

*Proof In *the case that *C *is a circle, *C *(*s*) *= *(cos(*s*); sin(*s*)). Due to the properties of functions *sine *and *cosine*, we considered just the case when *p = *(*x*, 0), with *−*1 *< x <*1. For the others values, we can simply rotate the coordinate system or get them by symmetries. Applying the symmetry once more, *n_y _*(*p*) must be zero, since the contribution of *C *(*s*) is canceled by the contribution of *C *(*−s*). After performing the integration, we conclude:

$$w\left(p\right)=\int_{0}^{2\pi }\frac{ds}{{\left(x-\text{cos}\left(s\right)\right)}^{2}+{\text{sin}}^{2}\left(s\right)}=\frac{2\pi }{1-x^{2}},$$

and

$$n_{x}\left(p\right)=\int_{0}^{2\pi }\frac{\text{cos}\left(s\right)ds}{{\left(x-\text{cos}\left(s\right)\right)}^{2}+{\text{sin}}^{2}\left(s\right)}=\frac{2\pi x}{1-x^{2}}.$$

As n_y _(*p*) *= *0 by the symmetry, ${\text{n}}_{\text{z}}\left(p\right)=\sqrt{1-x^{2}}$. Therefore, the normal field is exactly the same of a sphere of radius 1 centered at the origin.

When we consider internal curves to interpolate the normals, points on these curves are presented as singularities on the surface. On the other hand, if we consider only the boundary, the surface behaves smoothly over the domain.

**Figure 5:**
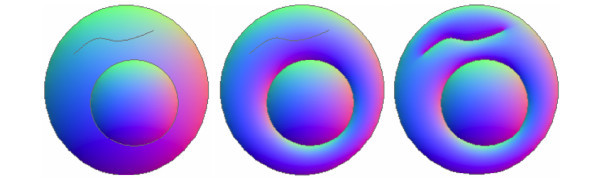
**Only the boundary of each region affect the interpolation normals (left)**. The boundary of the smallest circle also affected the exterior region (middle). Both the boundary of the smallest circle and the internal curve also affected the normal map (right).

Unlike Johnston [14], where an approximated normal propagation is obtained to calculate the normal map, our explicit formulation is accurate. Moreover, due to the flexibility of the proposed structure, it is possible to choose which curve contributes in the region interpolation process. This makes it possible to obtain effects as shown in Figure 5.

### Normal operators

The proposed region-tree structure is also suitable for applying attribute operators to the regions and to the curves of the cartoon. As the flexibility of our region-tree allows quick access to topological structures present in the cartoon, we can modify, in an efficient and independent way, the attributes of curves and regions. Some attributes are color, thickness, normals, depth, etc. To demonstrate this flexibility, we implemented two normal operators: *the scale region operator*, and the *depth curve operator*.

#### Scale operator

**Figure 6:**
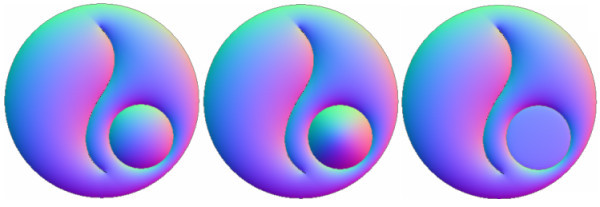
**Scale Region Operator: Normalized normal mapping (left)**. Factor 5.0 *z*-scale applied to the smallest circle (middle). Factor 0.1 *z*-scale applied to the smallest circle (right).

Given a region *R*, the operator consists in scaling the *n_x_, n_y_*, or *n_z _*normal coordinate. If the scale is applied on the *n_z _*coordinate, the visual effect is to lift the curve when illumination is applied. Figure 6 shows two different *z*-scales applied to the normal mapping of the smallest circle.

#### Depth operator

This operator deals with the idea of rising/sinking the selected curve *C *located in a region. The user controls the affected area surrounding the curve *C *and may rise or sink this curve. To do so, we calculate the distance information of each pixel located in a tubular neighborhood of *C *whose radius is the maximum distance *d_max _*chosen by the user. All pixels located in the tubular region will have their normal affected by the depth operator. For each pixel *p_i_*, with normal *n*(*pi*), we obtain its distance *d*(*pi*) to the curve and calculate its new normal *n*(*pi*) by adding an increase vector *u*(*pi*) to the vector *n*(*pi*). The increase vector *u*(*pi*) is calculated by some function considering that it converges to (0, 0, *K *) if *p_i _*is near to the curve, and converges to (0, 0, 0) if vector *p_i _*is away from the curve. We implemented the following equation:

$$\bar{n}(p_{i})=\left(1-\varphi \left(\frac{d(p_{i}}{d_{max}}\right)\right)(k-n(p_{i}))+\left(n(p_{i})\varphi \left(\frac{d(p_{i})}{d_{max}}\right)\right).$$

Where, $\bar{n}\left(p_{i}\right)$ is the new normal vector of the point *p_i_*, *n*(*pi*) is the normal vector of the point *p_i_, k = *(0, 0, 1) and *φ *is a function where *φ*(0) = 0 and *φ*(1) = 1. We used

$$\varphi \left(x\right)=\frac{-cos\left(\pi x\right)+1}{2}$$

**Figure 7:**
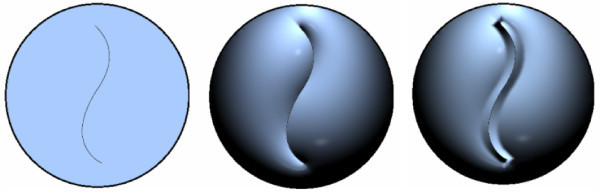
**Depth Curve Operator: Initial region (left)**. Interpolated normal mapping (middle). Depth operator applied to the internal curve (rigth).

Figure 7 shows the depth operator applied to a sphere.

## Automatic colorization across a frame sequence

The process of coloring manually each frame in a cartoon animation sequence is one of the most laborious steps. This process requires extensive work by the artist, both due to the large number of frames as the amount of detail in each one of them.

We present an automatic colorization technique based on our region-tree representation. The region-tree of consecutive frames allow the color transfer along the cartoon sequence.

If during the animation the region-tree is changed (regions appear/ disappear) a backtrack is performed to recover the information.

### Regions tracking

The first step to enable an automatic colorization is to find out the correspondence from consecutive frames. One way to obtain the best associations is to perform a regions tracking between both region-tree. In general, those frames present small variations throughout the scene, therefore, the association can be performed by analyzing parameters like position, area, shape and topology [4].

Let *R_i _*be a region with *p_i_, 0 ≤ i ≤ n *boundary points. The local area *A *associated to each *p_i _*is calculated by:

$$A=\frac{1}{2}\overset{n-1}{\underset{i=0}{\sum }}\left(x_{i}\cdot y_{i+1}-x_{i+1}\cdot y_{i}\right).$$

The region position is represented by its centroid *C = *(c_x_, C_y_), where

$$c_{x}=\frac{1}{6A}\overset{n-1}{\underset{i=0}{\sum }}\left(x_{i}+x_{i+1}\right)\left(x_{i}\cdot y_{i+1}-x_{i+1}\cdot y_{i}\right),$$

and

$$c_{y}=\frac{1}{6A}\overset{n-1}{\underset{i=0}{\sum }}\left(y_{i}+y_{i+1}\right)\left(x_{i}\cdot y_{i+1}-x_{i+1}\cdot y_{i}\right).$$

### Adjacency graph and neighborhood function

The *adjacency graph *is utilized in this work as way to make the region's tracking in a consistent way.

**Figure 8:**
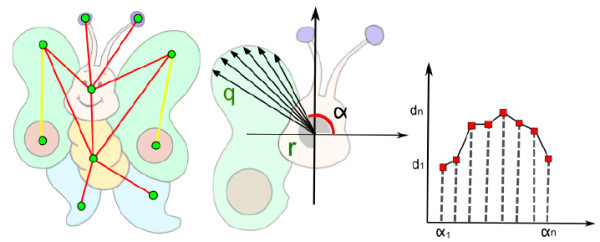
**Adjacency graph (left)**. Scheme to obtain the neighborhood function for a region *R*, where *α *= tan(*a*) (middle). Neighborhood function of *R *(right).

The graph is extracted from the region-tree by looking for regions that satisfy two conditions: sharing of edges and inclusion relationship. In Figure 8 (left) the adjacency graph is shown, where the yellow edges represent inclusion relationships and red ones represent regions sharing the same boundary.

Let *R *be a region and *c_R _*its centroid. The neighborhood relationship between *R *and its neighbor *Q *with boundary points *p_Qj_*, 1 ≤ *j *≤ *m*, is given by a neighborhood function *nF_RQ_*:

$$nF_{RQ}:=\left(\alpha_{RQ},d_{RQ}\right),$$

where

$$\begin{array}{ll} d_{RQ} & =\text{max }\left\{c_{R},\;dist\left(p_{Qj}\right)\right\},\;\\ & \;1\leq j\leq m\;\\ & \; \end{array}$$

α*_RQ _*is the slope of the line from *c_R _*to

$$\begin{array}{l} \text{arg }\max\;c_{R},dist(p_{Qj}).\\ 1\leq j\leq m \end{array}$$

arg max *c_R_,dist *( *p_Qj _*).

1*≤ j ≤m*

So, if an image's region *R *has *n *neighbors, it will have *n *neighborhood functions (see Figure 8 (right)).

### Associations between consecutive frames

The simplest way to create associations between regions from two consecutive frames could be comparing all the vertices from both frames graph. However, this option is also computationally expensive. To solve this problem, we propose the use of a region-tree in order to reduce the cost.

We execute a breadth-first search in the source-graph and destiny-graph initialized from the image background, since this is the only region of occurrence guaranteed in all images. In each search iteration, we analyze parameters of the area, position, contours, and neighborhood between regions represented by the compared vertices. The neighborhood parameter is defined by the *equivalence degree *(*ED*) from two regions.

**Figure 9:**
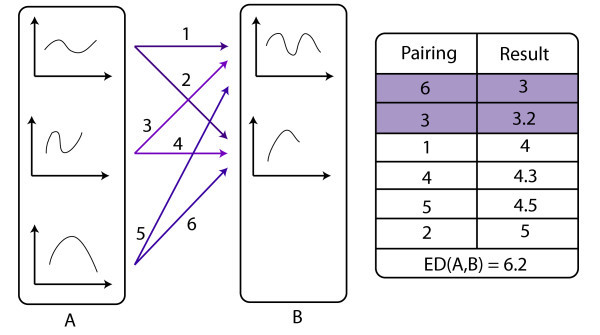
**Equivalence Graph: The equivalence degree ED of *A *and *B *is obtained by the sum of two smallest quadratic difference between its neighborhood function: ED(A,B) = 3 + 3.2 = 6.2**.

As shown in the picture bellow, the ED between a region *A *from frame *i *and *B *from frame *i *+ 1, where A and B have three and two neighboring regions respectively, is given by the sum of two smallest quadratic difference between its neighborhood function (see Figure 9 right).

**Figure 10:**
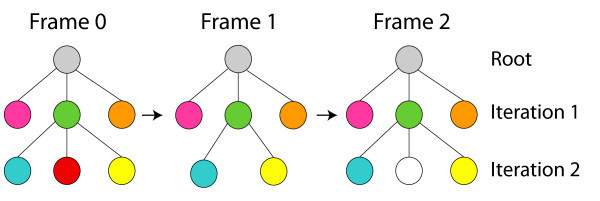
**Automatic Colorization: In the Iteration 2, the red region disappeared in the second graph *i.e.*, the region was occluded**. Therefore, this region color is discarded along the animation.

If the number of regions in the destiny-graph, in the current iteration, is bigger than in the source-graph, these regions are taken as new regions in the animation and they are not colored Figure 10. Otherwise, the region color in the source-graph is discarded along the animation process.

### Recursive regions tracking - recovering occluded regions

In the animation process, there are cases where a region can disappears in a frame and shows up in another one in the sequence. This happens every time that we have occlusion between regions during the animation. For those cases, the regions tracking (Section 4.1) is unsatisfying for the colorization process. Our work proposes tracking in a recursive way to improve the process by recovering the information of these occluded region.

In the recursive regions tracking, whenever a color information is passed from a region *A *to a region *B *in the consecutive frame, the vertex relative to the region A is marked. Once the frame is colorized, the algorithm verifies if there is any region not colorized in the frame. If all regions are colorized, the process will continue making a better association between frames as described in Section 4.3.

For the case where there is a not colorized region, the process is temporarily interrupted. Through the images adjacency graph, we find out which are the *n *neighboring regions of the non-colored one. In this case a reverse search is done through previous frames. In each analyzed frame, we look for regions corresponding to the *n *regions we identified. Once the corresponding regions are found, we check if they are unmarked neighboring regions. If this occurs, the unmarked regions are stored in a list of possible candidates to be equivalent to the non-colorized region. These candidates are collected in each frame.

**Figure 11:**
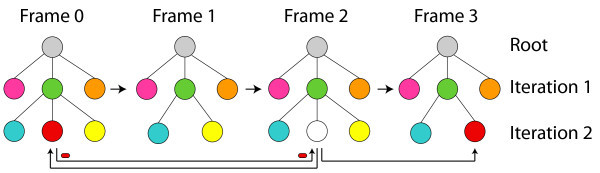
**Automatic Recursive Colorization: If a region disappeared, *i.e*., the number of regions in the destiny-graph is bigger than in the source-graph, a reverse search through previous frames is made**.

After the search, the algorithm goes back to the frame where the process stopped. All the candidates are analyzed by the same parameters: area, contour, position, and equivalence degree. An association between the best candidate and the non-colorized region is created passing all color information. If two or more candidates are selected, no association is created and the region is not colorized. Figure 11 illustrates the recursive colorization process, where the color of the white region is recovered from the first frame and then is passed to consecutive frames.

## Results and discussion

**Figure 12:**
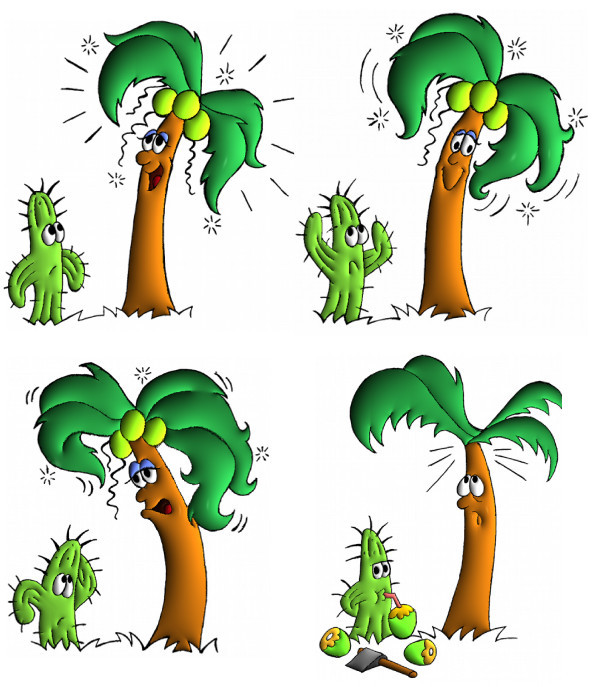
**Colorization and Illumination from a free-hand sketch**.

This section presents some results of our automatic colorization and illumination technique. From a free-hand sketch drawing, a cartoon sequence could be colorized and than illuminated creating a 3D effect for the drawing (see Figure 12). In this work, we applied to all results a *phong *illumination [13], although with the normal mapping (Section 3) any illumination effect could be performed.

**Figure 13:**
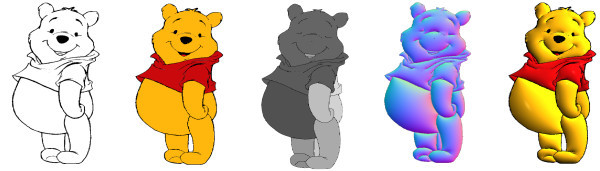
**Illumination Process: From left: original image, colorized cartoon, segmented cartoon, normal map, illuminated cartoon**.

**Figure 14:**
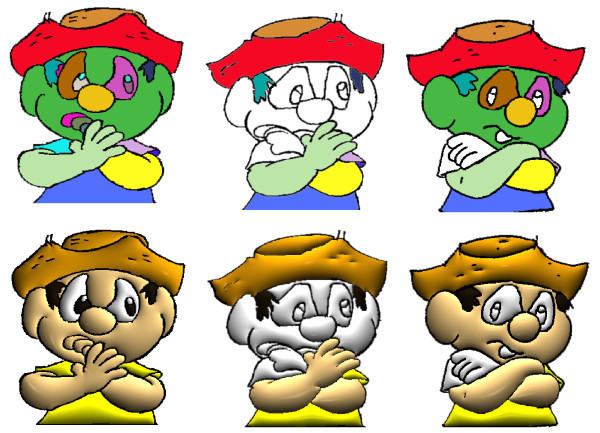
**From left: segmented cartoon, split of two regions (face), recovery of the color**.

In Figure 13 is shown the main steps of the illumination process. From a black and white cartoon as input, the user provide the color information that will be used in the illumination. The algorithm perform the image's segmentation and computes the normal map which will be used to create the 3D effect. In Figure 14 the *Recursive Region Tracking *(Section 4.4) is performed to recover the color information that was lost during the animation. In this sequence the model's face (the green area) was split in two different regions changing the adjacency graph structure since ne1w nodes are inserted.

These regions are new in the frame so the algorithm does not recognize them. In the recursive tracking, when the character's face comes back to being one region representation the algorithm allows recover its color by matching its adjacency graph with the first one.

**Figure 15:**
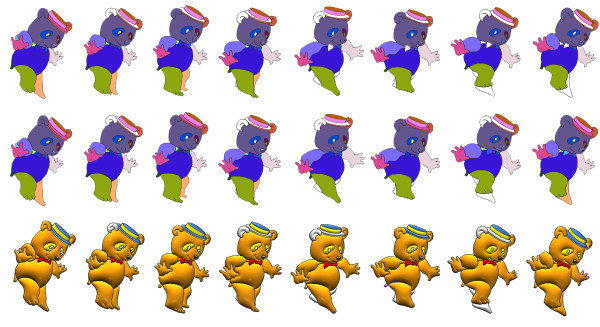
**The first sequence shows the method from Bezerra et al**. [4]. The second sequence shows our proposed method of colorization. The third sequence shows our illumination process.

**Figure 16:**
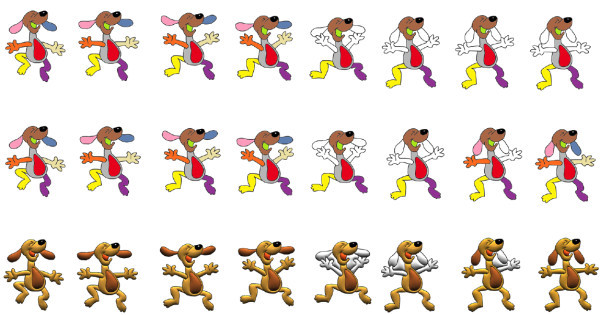
**The first sequence shows the method from Bezerra et al**. [4]. The second sequence shows our proposed method of colorization. The third sequence shows our illumination process.

Figures 15 and 16 show some comparisons between our recursive colorization method based on a region-tree representation and the colorization method proposed by Bezerra et al. [4].

In these cases, we can observe that meaningful changes in the adjacency graph resulting from occlusion and/or creation of regions may cause less impact on the sequence if the recursive tracking is performed. Once the color information is lost, methods like Bezerra et al. [4] are not able to recover the informations causing many frames without proper colorization.

Figure 15 shows the occlusion problem in the bear's left leg. In the first image sequence, the color information cannot be recovered since this information is lost in the fifth image because there was a significant topological change such as a region splitting into two. Secondly regions having its neighborhood drastically altered such as the left eye, the hats brim, and the tie are lost in Bezerra et al. [4] tracking. In the case of the eye, it occurs when the eye links to the bear's face region. In the hats brim case, it occurs when the brim is linked to a new region the bear's ear. Finally, in the tie case, it occurs because one region disappears joining the bear's arm.

In Figure 16 we do not have a region occlusion. On the other hand, when the dog's hands touches his ears two regions are created changing the adjacency graph. Since these regions are new the algorithm do not colorize them. In the first sequence, once the color is lost, the method from Bezerra et al. [4] is not able to colorize the following frames. The recursive method however (second sequence), allows that the color information is propagated from previous frames making possible the gradual and complete cartoon's colorization (far right image).

**Figure 17:**
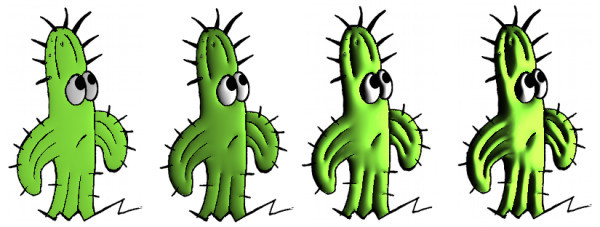
**Scale Operator: From left the values are: 0.1, 0.5, 2.0, 10.0**.

The Figure 17 illustrates the result of the scale operator. We used different scalar factor values to illustrate the effect in our cartoon.

### Limitations

Our method present some limitations both in the method of normal vector calculation as in the automatic colorization.

In the normal map calculation, if a point belongs to a curve, its *z*-component is 0. If this curve is interior to a region, our interpolation scheme will create singularities in the surface. It would be necessary to define normal operators to avoid these singularities.

**Figure 18:**
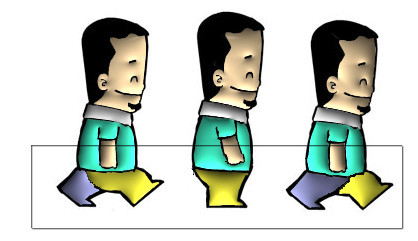
**Problem with the tracking in walk motion**.

In the automatic colorization, a problem could occurs in situations where the regions are similar, e.g., in a walk motion of a character. The two legs of the character are topologically identical, they have identical area, position and neighborhood. Then, in the motion, when the left leg surpassing the right one, the algorithm swaps the colors of both, since the position of the left leg is replaced by similar topological information right leg. Figure 18 shows this problem.

Another limitation is that illumination is not transferred frame by frame. It is computed separately in each frame.

## Conclusion and future works

This work presented a new strategy based on a region-tree structure to represent 2D drawings. Unlike usual structures, our representation scheme explores both the time coherence of the topological structures, as well as the local spatial information of each frame. Therefore our method is both suitable for colorization and to illumination process.

Due to the topological and geometrical information of this region-tree representation, we developed a method for approximating lighting to 2D drawings: we compute the normal mapping of the drawing based on our new topological structure. Our illumination method is suitable for independently shading parts of the drawing, like curves and regions.

The topological structure inspired a colorization method, where color is transferred recursively during the animation. With the recursive tracking method, we can recover most of the lost regions in the animation.

Future works include: verify time coherence together with spacial local structure to transfer frame by frame illumination; define new attribute operators; optimize the normal interpolation, by avoiding that invisible curve points affect the resulting normal inside the region.
